# Supplementary material for: Analysis of PPARγ Signaling Activity in Psoriasis
Source: Int J Mol Sci. 2021 Aug 10;22(16):8603. doi: 10.3390/ijms22168603 (PMC8395241; doi:10.3390/ijms22168603)
Supplement: Supplementary file 1 [file ijms-22-08603-s001.zip › Supplemental materials_Analysis of PPARg signaling activity in psoriasis/Pathway models/Models images and html files/Model of PPARG related pathways in psoriasis (short version)/160670.html]

Gb/g


# Functional Class Gb/g

|  |  |
| --- | --- |
| URN | urn:agi-aopfc:0000536 |
| Total Entities | 2 |
| Connectivity | 1499 |
| Name | Gb/g |
| Description | G beta/gamma |

---

|  |  |
| --- | --- |
| ChildConcepts | G-beta |
|  | G-gamma |

---

|  |  |
| --- | --- |
| Pathway | Melatonin Effects on Circadian Cycle |
|  | High Fat Diet Activates cAMP Related Exocytosis in Synapse and Endocrine Cell |
|  | GCG and PPY Regulate Metabolism and Satiety |
|  | Ghrelin and Nesfatin-1 Regulate Stomach Functional State and Satiety |
|  | mTOR Signaling Activation by Taste Sensor Receptors (Rodent Model) |
|  | Ghrelin and SST Regulate GH1 Release in Hypothalamus |
|  | Taste Receptors Activation in Tongue |
|  | L-cell: GCG, PYY and 5-HT Release |
|  | I-cell: CCK-8 Secretion and Regulation of Eating Behavior |
|  | A-cell (X-like): Ghrelin and Nesfatin-1 Synthesis |
|  | G-cell: Gastrin Secretion |
|  | N-cell: Neurotensin Production |
|  | Dopamine Receptors Signaling |
|  | Serotonin Receptors Signaling |
|  | CXC Chemokine Receptor Signaling |
|  | CC Chemokine Receptor Signaling |
|  | Proplatelet Maturation |
|  | Platelet Activation via GPCR Signaling |
|  | Leukotriene Effect on Vascular Endothelial Cell Response |
|  | Eicosanoids in Myeloid Cell Activation |
|  | Leukotriene Effect on Airway Smooth Muscle Response |
|  | Histamine Receptor H3 Signaling in Neurons |
|  | Neutrophil Activation via LTB4/Polyisoprenyl Phosphate Signaling |
|  | Basophil Activation |
|  | Eosinophil Activation |
|  | Mast-Cell Activation without Degranulation through CYSLTR1/CYSLTR2 Signaling |
|  | Mast-Cells De Novo Synthesized Mediators via IgE Independent Signaling |
|  | Neutrophil Recruitment and Priming |
|  | Neutrophil Degranulation via FPR1 Signaling |
|  | CR3 Mediated Phagocytosis in Neutrophils and Macrophages |
|  | Neutrophil Chemotaxis |
|  | Neutrophil Activation via Adherence on Endothelial Cells |
|  | Vascular Endothelial Cell Activation by NO |
|  | Vascular Endothelial Cell Activation by Cytokines |
|  | Lipolysis Regulation in Adipocyte |
|  | Potassium Flux Regulation |
|  | Nociception Expression Targets Signaling |
|  | Sodium Flux Regulation |
|  | Vascular Motility |
|  | Ca2+ Flux Regulation |
|  | TAAR1 -> Neurotransmitter Uptake |
|  | NPY1R -> CRH/POMC Production |
|  | EDNRA/B -> Vascular Motility |
|  | ADORA1/2A -> Exocytosis |
|  | ADORA3 -> Mast-Cell Degranulation |
|  | ADRA2A -> Neurotransmitter Release |
|  | ADRB1 -> Prostaglandin Generation |
|  | BDKRB1/2 -> Vasodilation |
|  | BDKRB1/2 -> Ion Channels |
|  | BDKRB1/2 -> Prostaglandin Generation |
|  | BDKRB1/2 -> Interleukins Production |
|  | CNR1/2 -> Membrane Transport |
|  | CNR1/2 -> Vascular Motility |
|  | CNR1/2 -> IL1B/2/4/6/10 Production |
|  | DRD3 -> Dopamine Uptake |
|  | DRD2 Expression Targets |
|  | DRD1/3 -> Potassium Uptake |
|  | DRD2/4 -> Membrane Transport |
|  | GALR1/2/3 -> POMC/NPY Production |
|  | HRH1/2 -> Vascular Motility |
|  | HRH2/4 -> IL6/10 Production |
|  | CHRM1 -> IL2 Production |
|  | CHRM1/2/3/5 -> Ion Channels |
|  | CHRM1/2/3 -> Vascular Motility |
|  | OPRD/OPRM -> Ion Channels |
|  | OPRK -> Pain Perception |
|  | OPRL1 -> Ion Channels |
|  | PTGER2/3 -> Vascular Motility |
|  | PTGER2/3 -> Inflammation-Related Expression Targets |
|  | HTR1 -> IL6 Production |
|  | HTR5 -> TNF Production |
|  | HTR2 -> Membrane Transport |
|  | HTR1 -> Membrane Transport |
|  | HTR1 -> Vascular Motility |
|  | TACR1 -> TNF/IL6/IL8 Production |
|  | GABA(B)R -> Postsynaptic Inhibition |
|  | GRM1/5 (Postsynaptic) -> Ion Channels |
|  | P2RY2/12/13/14 -> N-Type Calcium Channel |
|  | P2RY1/2/4/6 -> Potassium Channels |
|  | Melatonin in Cell Survival and Antioxidant Response |
|  | Circadian Clock Genes in Suprachiasmatic Nuclei Neurons |
|  | Hypocretin (Orexin) in Arousal Regulation |
|  | Dilated Cardiomyopathy |
|  | Cardiomyocyte Hypertrophy |
|  | Hypertrophic Cardiomyopathy |
|  | Endothelial Cell Dysfunction in Pulmonary Hypertension |
|  | Smooth Muscle Cell Dysfunction in Pulmonary Hypertension |
|  | Vascular Reactivity in Raynaud Disease |
|  | NO Synthesis in Early Vasospasm |
|  | Contraction Due Vasospasm |
|  | Melanoma |
|  | MITF as a Regulator of Melanoma Cell Development |
|  | Oxidative Stress in Vitiligo |
|  | Vitiligo |
|  | G-Proteins Signaling in Insulin Secretion in beta-Cell |
|  | Metastatic Colorectal Cancer |
|  | Antiphospholipid Antibodies in Platelets and Coagulation Cascade |
|  | Platelet and Coagulation Cascade Activation by Antiphospholipid Antibodies |
|  | Eosinophil Activation and Degranulation in Asthma |
|  | Airway Smooth Muscle Cell High Rate Proliferation |
|  | Airway Smooth Muscle Cell Contraction |
|  | Neutrophil Degranulation via FPR1/IL8 |
|  | Basophil Activation in Asthma |
|  | Mast-Cell Related Chemokines Signaling in Asthma |
|  | Skin Fibrosis |
|  | ADK Expression Upregulation |
|  | CCL2 Role in CGRP Expression and Release |
|  | Vascular Smooth Muscle Cell Vasodilation in Brain |
|  | NO Effect on Vasodilation and Vasoconstriction |
|  | Hypocretin Elevation and Monoamine Oxidase Decrease/Inhibition |
|  | Sleep Disturbance (Hypothesis) |
|  | MIF Role in Cholesteatoma |
|  | Cholesteatoma |
|  | Prostate Cancer |
|  | Muscarinic Receptor-Mediated Airway Smooth Muscle Contraction |
|  | Penile Detumescense Augmentation Mechanisms |
|  | Penile Erection Impairment |
|  | Noradrenaline/Gs Expression Targets |
|  | Epinephrine/Gs Expression Targets |
|  | Acetylcholine Expression Targets |
|  | Dopamine/Gi Expression Targets |
|  | CXCL6 Expression Targets |
|  | CXCL1 Expression Targets |
|  | CXCL2 Expression Targets |
|  | CXCL3 Expression Targets |
|  | CXCL5 Expression Targets |
|  | Complement Component GPCRs Signaling |
|  | Clozapine Induced Granulocytopenia |
|  | Erythropoietin -> NF-kB Expression Targets |
|  | Erythropoietin -> ELK/SRF Expression Targets |
|  | FPR1 -> Cytoskeleton Signaling |
|  | FSHR Expression Targets |
|  | Glutamate/Gq Expression Targets |
|  | EGFR Transactivation in Cancer and Non-Cancer Cells |
|  | IGF2 -> HIF1A/MYC Expression Targets |
|  | IGF1 -> ELK/SRF/HIF1A/MYC/SREBF Expression Targets |
|  | Insulin -> ELK/SRF/HIF1A/MYC/SREBF Expression Targets |
|  | IGF1R -> ELK/SRF/HIF1A/MYC/SREBF Signaling |
|  | Thromboxane A2 Expression Targets |
|  | LPA Expression Targets |
|  | Dronabinol/Anandamide Expression Targets |
|  | S1P Expression Targets |
|  | MTNR1 Signaling |
|  | EDN1 Expression Targets |
|  | NPY Expression Targets |
|  | EDN3 Expression Targets |
|  | Lysophosphatidic Acid/LPARs Signaling |
|  | Ras-GRF Regulation Signaling |
|  | PI3K/RAC1 Signaling |
|  | IL1B -> PGE2 Expression Targets |
|  | ace2 |
|  | Model of PPARG signaling in psoriasis |
|  | Model of PPARG related pathways in psoriasis (short version) |
|  | 1\_Formation of cysts in polycystic kidney disease |
|  | 1\_Endothelial cells stimulate vasoconstriction and thrombosis in pulmonary hypertension |
|  | 2\_Cardiomyocyte hypertrophy |
|  | 2\_The intense proliferation and contractile activity of smooth muscle cells in pulmonary hypertension |
|  | 2\_1\_Eosinophilia and neutrophilia in asthma: eosinophil migration and activation |
|  | 4\_1\_Airway smooth muscle cells contraction |
|  | 1\_2\_Insulin synthesis and secretion: insulin secretion |
|  | 1\_1\_Insulin synthesis and secretion: insulin processing and storage |
|  | 1\_Decreased secretion of thyroid hormones |
|  | Model of PPARG signaling in psoriais (tested) |

---

|  |  |
| --- | --- |
| MedScan ID | 12100266 |

---

|  |  |
| --- | --- |
| Alias | Getagamma |
|  | G-protein beta/gamma-subunit complex |
|  | beta-gamma complexes |
|  | G(beta)(gamma) heterodimer |
|  | G-beta/G-gamma complexes |
|  | G-beta/G-gamma complex |
|  | G bg |
|  | G(beta)(gamma) |
|  | G-protein beta/gamma-subunit complexes |
|  | beta-gamma complex |
|  | Gb/g |
|  | G(beta)(gamma) heterodimers |
|  | G protein bg |
|  | heterotrimeric G-protein GTPase, gamma subunits |
|  | heterotrimeric G-protein GTPase, gamma subunit |
|  | heterotrimeric G-protein GTPase, beta subunit |
|  | heterotrimeric G-protein GTPase, beta subunits |
|  | G b/g |
|  | G protein b/g |
|  | G protein beta gamma |
|  | G protein beta/gamma |

---

|  |  |
| --- | --- |
| Cell Localization | Cytoplasm |

---
